# Effects of SARS-COV-2 infection on outcomes in patients hospitalized for acute cardiac conditions. A prospective, multicenter cohort study (Swiss Cardiovascular SARS-CoV-2 Consortium)

**DOI:** 10.3389/fcvm.2023.1203427

**Published:** 2023-10-13

**Authors:** Konstantinos C. Koskinas, Raphael Twerenbold, David Carballo, Christian M. Matter, Stephane Cook, Dik Heg, Andre Frenk, Stephan Windecker, Stefan Osswald, Thomas F. Lüscher, Francois Mach

**Affiliations:** ^1^ Department of Cardiology, Bern University Hospital Inselspital, Bern, Switzerland; ^2^ Department of Cardiology, Basel University Hospital, Basel, Switzerland; ^3^ University Center of Cardiovascular Science & Department of Cardiology, University Heart and Vascular Center Hamburg, University Medical Center Hamburg-Eppendorf, Hamburg, Germany; ^4^ German Center for Cardiovascular Research (DZHK) Partner Site Hamburg–Kiel–Lübeck, Hamburg, Germany; ^5^ Division of Cardiology, Geneva University Hospitals, Geneva, Switzerland; ^6^ Department of Cardiology, Zurich University Hospital, Zurich, Switzerland; ^7^ Department of Cardiology, Fribourg Canton Hospital, Fribourg, Switzerland; ^8^ CTU Bern, University of Bern, Bern, Switzerland; ^9^ Department of Cardiology, Royal Brompton & Harefield Hospitals and National Heart and Lung Institute, Imperial College, London, United Kingdom; ^10^ Center for Molecular Cardiology, University of Zurich, Zurich, Switzerland

**Keywords:** COVID-19, pandemic, cardiovascular, prognosis, mortality

## Abstract

**Background:**

Although the severe acute respiratory syndrome coronavirus 2 (SARS-COV-2) causing coronavirus disease 2019 (COVID-19) primarily affects the respiratory system, the disease entity has been associated with cardiovascular complications. This study sought to assess the effect of concomitant SARS-COV-2 infection on clinical outcomes of patients hospitalized primarily for acute cardiac conditions on cardiology wards in Switzerland.

**Methods:**

In this prospective, observational study conducted in 5 Swiss cardiology centers during the COVID-19 pandemic, patients hospitalized due to acute cardiac conditions underwent a reverse-transcriptase polymerase chain reaction test at the time of admission and were categorized as SARS-COV-2 positive (cases) or negative (controls). Patients hospitalized on cardiology wards underwent treatment for the principal acute cardiac condition according to local practice. Clinical outcomes were recorded in-hospital, at 30 days, and after 1 year and compared between cases and controls. To adjust for imbalanced baseline characteristics, a subgroup of patients derived by propensity matching was analyzed.

**Results:**

Between March 2020 and February 2022, 538 patients were enrolled including 122 cases and 416 controls. Mean age was 68.0 ± 14.7 years, and 75% were men. Compared with controls, SARS-COV-2-positive patients more commonly presented with acute heart failure (35% vs. 17%) or major arrhythmia (31% vs. 9%), but less commonly with acute coronary syndrome (26% vs. 53%) or severe aortic stenosis (4% vs. 18%). Mortality was significantly higher in cases vs. controls in-hospital (16% vs. 1%), at 30 days (19.0% vs. 2.2%), and at 1 year (28.7% vs. 7.6%: *p* < 0.001 for all); this was driven primarily (up to 30 days) and exclusively (at one-year follow-up) by higher non-cardiovascular mortality, and was accompanied by a greater incidence of worsening renal function in cases vs. controls. These findings were maintained in a propensity-matched subgroup of 186 patients (93 cases and 93 controls) with balanced clinical presentation and baseline characteristics.

**Conclusions:**

In this observational study of patients hospitalized for acute cardiac conditions, SARS-COV-2 infection at index hospitalization was associated with markedly higher all-cause and non-cardiovascular mortality throughout one-year follow-up. These findings highlight the need for effective, multifaceted management of both cardiac and non-cardiac morbidities and prolonged surveillance in patients with acute cardiac conditions complicated by SARS-COV-2 infection.

## Introduction

Coronavirus disease 2019 (COVID-19), caused by the severe acute respiratory syndrome coronavirus 2 (SARS-CoV-2), was declared by the World Health Organization a pandemic in March 2020 and has continued to cause outbreak waves globally. Although clinical manifestations of SARS-CoV-2 predominantly consist of respiratory symptoms, numerous patients with COVID-19 present with cardiovascular conditions or may develop cardiovascular complications during the course of the disease (1, 2). During the early outbreak in China, cardiovascular rather than respiratory symptoms were reported as initial symptoms by several patients with confirmed SARS-CoV-2 infection (3). Notably, myocardial injury manifested by elevated cardiac biomarkers had been frequently reported and associated with an unfavorable clinical course in patients with COVID-19 (4–6). Conversely, pre-existing cardiovascular disease (CVD), as well as cardiovascular risk factors (e.g., diabetes mellitus and arterial hypertension) have been identified as predictors of higher mortality in patients with COVID-19 (7–9). A comprehensive meta-analysis showed that a history of CVD tripled the odds of the occurrence of a severe course of COVID-19 (10).

Since the initial outbreak of the pandemic, extensive research has been able to demonstrate that COVID-19 shares many manifestations of a systemic disease with major implications for the cardiovascular system. A broad range of acute and longer-term cardiac complications of SARS-COV-2 infection have been reported (11) and mechanisms underlying COVID-19-related cardiac manifestations have been elucidated, providing important lessons for more effective management in the current, and possibly future pandemic crises. The aim of this study was to explore the impact of concomitant SARS-COV-2 infection on clinical outcomes in patients who were primarily hospitalized for acute cardiac conditions in Switzerland.

## Materials and methods

### Study design and study population

This was a prospective, multicenter, observational, case-control study aiming to evaluate in-hospital, 30-day, and one-year outcomes in SARS-CoV-2-infected vs. non-infected patients hospitalized for an acute cardiac condition in the Cardiology services (departments) of 5 Swiss hospitals. Patients hospitalized with cardiovascular conditions in other departments, notably, Intensive Care Unit or Internal Medicine, were not eligible for this cohort. There was no formal sample size calculation; rather, the number of study participants was based on a combination of feasibility and case prevalence at the participating sites. The study design followed the Strengthening the Reporting of Observational Studies in Epidemiology (STROBE) recommendations for observational studies and specific to the STROBE statement for Infectious Diseases (STROBE-ID) (12).

Beginning in March 2020, we prospectively enrolled patients aged >18 years admitted with one of the following cardiac diagnoses: (i) acute coronary syndrome (ACS) [ST-elevation myocardial infarction (STEMI) or non-ST-elevation acute coronary syndrome (NSTE-ACS)], diagnosed according to current guidelines (13, 14); (ii) new-onset major arrhythmia (atrial fibrillation, sustained or non-sustained ventricular tachycardia, ventricular fibrillation, high-degree atrioventricular block or clinically relevant bradyarrhythmia); (iii) acute or worsening heart failure (HF); (iv) symptomatic severe aortic stenosis; (v) hospitalization related to adult congenital heart disease; or (vi) clinically suspected acute myocarditis. Patients unable to understand the requirements of the study or unwilling to provide informed consent were excluded. All patients signed an informed consent form before any study-related evaluations. In addition to the prospective enrolment, a subsequent amendment of the initial protocol allowed for retrospective inclusion of patients who were identified through hospital medical record screening and met eligibility criteria, as long as general consent had been provided during index hospitalization or an informed consent form was signed subsequently for the purpose of this study. As per study protocol, the Steering Committee decided to terminate the study once the pandemic surges had elapsed according to national Swiss authorities, and the numbers of COVID-19-positive patients admitted to the Cardiology departments of the participating hospitals had minimized.

**Figure 1:**
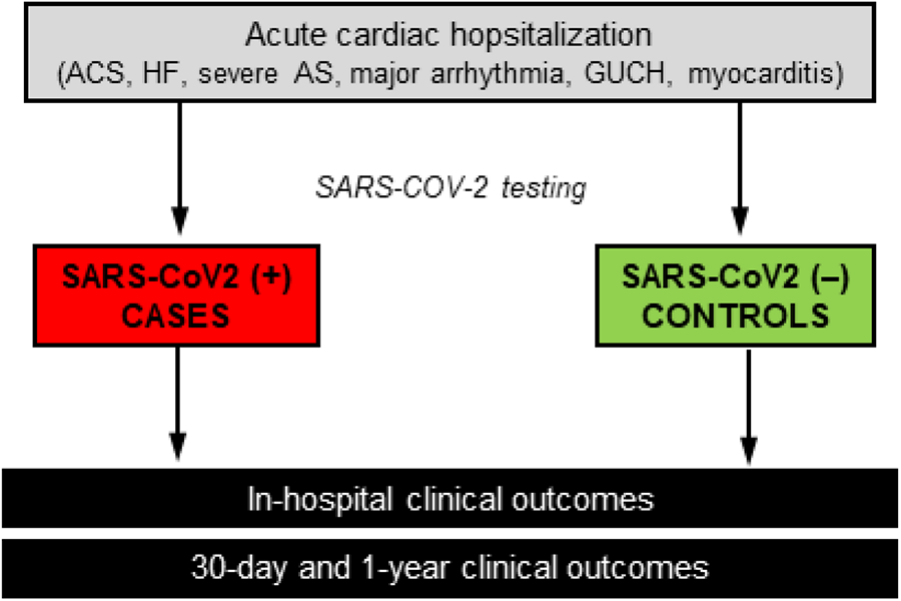
Summary of study flow.

As per institutional protocols across all participating centers, all patients who were admitted in the respective Cardiology departments during the enrolment period (irrespective of possible inclusion in the study) underwent a clinically indicated reverse transcription polymerase chain reaction (RT-PCR) test for SARS-COV-2 either immediately prior to admission, or as soon as reasonably possible after hospital admission. For the purpose of the study, enrolled patients who were SARS-CoV-2 positive comprised the group of cases, whereas all SARS-COV-2-negative patients comprised the control group (Figure 1).

Patients were treated for their acute cardiac conditions according to local institutional protocols, in line with current clinical practice guidelines. Treatment included medical therapy with or without clinically indicated interventions according to the underlying acute cardiac condition [e.g., coronary angiography with or without revascularization for patients presenting with ACS, or transcatheter aortic valve implantation (TAVI) for patients with severe symptomatic aortic stenosis].

Data on patient characteristics (demographics, comorbidities, medications, symptoms, vital signs) were collected from local patient data management systems. All patients underwent routine, clinically indicated, local laboratory testing that should include the following variables: complete blood count; high-sensitivity C-reactive protein (hsCRP); biomarkers of myocardial injury [creatine kinase (CK), CK-MB, high-sensitivity troponin-T (hs-TnT)]; and renal function testing [creatinine, estimated glomerular filtration rate (eGFR)]. These measurements were performed serially, at least twice during hospitalization; in cases with more than two measurements available, the one closest to hospital admission (“time point 1”) and the one closest to discharge (“time point 2”) were recorded for the study analyses; as an exception, the highest recorded (peak) values for biomarkers of myocardial injury were recorded in case of more than two available measurements.

### Patient follow-up

In-hospital clinical outcomes were assessed from discharge letters. For 30-day (±7 days) and 1-year (±30 days) follow-up, survival data were obtained from hospital records and municipal civil registries. A health questionnaire was sent by postal mail to all living patients with questions on rehospitalization and major cardiovascular events, followed by telephone contact in case of missing response. General practitioners, referring cardiologists, and patients were contacted as required for additional information. In case any potential clinical events were reported, hospital discharge letters were screened to define these events. In addition to mortality, outcomes of interest included myocardial infarction, unplanned coronary revascularization, cerebrovascular event (stroke or transient ischemic attack), acute kidney injury (defined as >2-fold increase in serum creatinine or >50% decrease in estimated glomerular filtration rate), major arrhythmia, and hospitalization for heart failure.

### Statistical analyses

This registry study was designed as a case-control study comparing outcomes between patients with vs. without SARS-CoV-2 infection, both in the whole cohort and within a matched cohort. No power analysis and sample size calculation were performed in this pragmatic, observational study. The date of the SARS-CoV-2 test result served as the index date for defining the exposure. We describe patient characteristics, performed procedures, and clinical outcomes in numbers and proportions [accompanied by 95% confidence intervals (CIs)]. For demographic and clinical characteristics, ANOVA was used to identify statistically significant differences in continuous variables, and the *χ*2 or Fisher's exact test in categorical variables. The matched cohort was based on propensity score matching (1:1) of cases vs. controls using a caliper of 0.2 and including age; sex; ACS; new-onset major arrhythmia; acute HF; diabetes; history of myocardial infarction; current smoking; chronic obstructive pulmonary disease; arterial hypertension; hypercholesterolemia; admission to Intensive Care Unit (ICU) during index hospitalization. These variables were pre-selected on the basis of known risk factors for mortality after COVID and intended to achieve also a good balance in the diagnostic groups. Clinical outcomes at 30 days and at 1 year since diagnosis were analysed using time-to-event, counts represent the first occurrence per patient, with the Aalen-Johanssen estimator of the cumulative incidence (%) under competing risk with all-cause mortality. Subdistribution ratio of the hazards using the Fine & Grey methodology (15) with 95% CI and *p*-value comparing SARS-CoV-2 positive (cases) vs. negative (controls) test result are provided. Statistical analyses were performed using Stata 17, and *p*-values <0.05 were considered significant.

## Results

Between March 16 2020 and February 27, 2022, 538 patients were enrolled: 122 SARS-COV-2-positive patients (cases; 22.7%) and 416 SARS-COV-2-negative patients (77.3%; controls).

### Baseline characteristics

Mean age was 68.0 ± 14.7 years, 75% of patients were men, and 29% had diabetes mellitus (Table 1). The most frequent diagnosis at index hospitalization was ACS (47%), followed by acute HF (21%), symptomatic severe aortic stenosis (15%), and major arrhythmia (14%). Diagnoses of clinically suspected myocarditis and hospitalization related to adult congenital heart disease were rare (2% and 1%, respectively). At the time of admission, 10% of patients had fever. During hospitalization, almost half of patients (46%) underwent invasive coronary angiography with or without intervention; 13% of patients underwent TAVI; and 8% underwent intervention for major arrhythmia. A total of 112 patients (21%) required subsequent admission to an ICU during index hospitalization.

**Table 1:** Baseline characteristics of enrolled patients.

	All patients *N* = 538	Controls *N* = 416	Cases *N* = 122	Difference (95% CI)	*p* value
Age (years)	68.0 ± 14.7	68.1 ± 14.7	67.6 ± 14.8	0.5 (−2.5; 3.5)	0.72
Male sex	406 (75%)	314 (75%)	92 (75%)	0	1.000
Diagnosis at index hospitalization					<0.001
Acute coronary syndrome	254 (47%)	222 (53%)	32 (26%)	27% (17%; 37%)	<0.001
New-onset major arrhythmia	75 (14%)	37 (9%)	38 (31%)	−22% (−29%; −15%)	<0.001
Acute heart failure	115 (21%)	72 (17%)	43 (35%)	−18% (−26%; −10%)	<0.001
Symptomatic severe aortic stenosis	79 (15%)	74 (18%)	5 (4%)	14% (7%; 21%)	<0.001
Hospitalization related to adult congenital heart disease	4 (1%)	3 (1%)	1 (1%)	−0% (−2%; 2%)	1.000
Clinically suspected myocarditis	11 (2%)	8 (2%)	3 (2%)	−1% (−3%; 2%)	0.72
Diabetes mellitus	154 (29%)	100 (24%)	54 (44%)	−20% (−29%; −11%)	<0.001
Current smoker	255 (47%)	205 (49%)	50 (41%)	8% (−2%; 18%)	0.12
Hypercholesterolemia	279 (52%)	225 (54%)	54 (44%)	10% (−0%; 20%)	0.06
Previous myocardial infarction	93 (17%)	67 (16%)	26 (21%)	−5% (−13%; 2%)	0.22
History of malignancy	83 (15%)	70 (17%)	13 (11%)	6% (−1%; 13%)	0.12
Renal insufficiency requiring dialysis	15 (3%)	11 (3%)	4 (3%)	−1% (−4%; 3%)	0.75
Chronic obstructive lung disease	57 (11%)	49 (12%)	8 (7%)	5% (−1%; 11%)	0.13
Clinically relevant valvular disease	86 (16%)	75 (18%)	11 (9%)	9% (2%; 16%)	0.02
Type of aortic valvular disease	n = 83	n = 75	n = 8		0.09
Severe native aortic valve stenosis	71 (86%)	66 (88%)	5 (63%)	26% (0%; 51%)	0.09
Degenerated aortic bioprosthesis	6 (7%)	5 (7%)	1 (13%)	−6% (−25%; 13%)	0.47
Severe aortic regurgitation	6 (7%)	4 (5%)	2 (25%)	−20% (−38%; −1%)	0.101
NYHA class					<0.001
NYHA I	222 (42%)	198 (48%)	24 (22%)	26% (16%; 36%)	<0.001
NYHA II	103 (20%)	85 (21%)	18 (17%)	4% (−4%; 12%)	0.42
NYHA III	105 (20%)	88 (21%)	17 (16%)	6% (−3%; 14%)	0.23
NYHA IV	93 (18%)	43 (10%)	50 (46%)	−35% (−43%; −28%)	<0.001
Cardiogenic shock (Killip 4)	25 (5%)	12 (3%)	13 (11%)	−8% (−12%; −4%)	0.001
Fever	56 (10%)	22 (5%)	34 (29%)	−24% (−29%; −18%)	<0.001
Interventions performed during index hospitalization					
Coronary procedure	249 (46%)	223 (54%)	26 (21%)	32% (23%; 42%)	<0.001
TAVI	72 (13%)	70 (17%)	2 (2%)	15% (8%; 22%)	<0.001
Intervention for major arrhythmia	44 (8%)	31 (7%)	13 (11%)	−3% (−9%; 2%)	0.26
Transfer to intensive care unit during index hospitalization	112 (21%)	52 (13%)	60 (49%)	−37% (−44%; −29%)	<0.001

Depicted are means with standard deviations (±) and counts with percentages of patients with non-missing data (%). *P*-values from *t*-tests or Fisher's tests (- or chisquare tests in case more than 2 × 2 comparisons). NYHA, New York Heart Association; TAVI, transcatheter aortic valve index.

**Figure 2:**
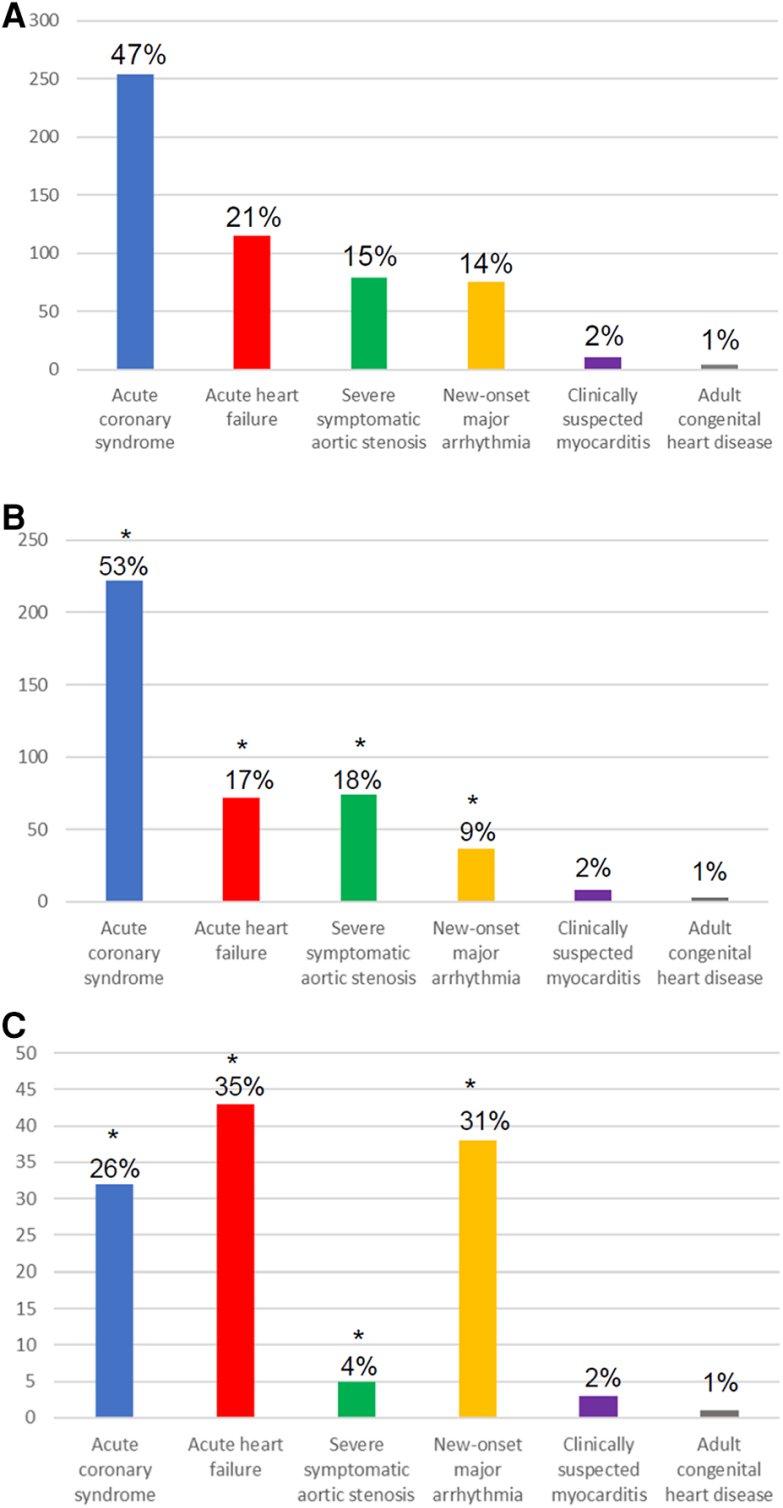
Diagnosis at index hospitalization among all patients (**A**); SARS-COV-2-negative patients (**B**); and SARS-COV-2-positive patients (**C**). * indicates statistically significant difference (*p* < 0.05) between the SARS-COV-2-positive and SARS-COV-2-negative group.

Patients with SARS-COV-2 infection, as compared with controls, more frequently presented with acute HF (35% vs. 17%) or major arrhythmia (31% vs. 9%), and less commonly with an ACS (26% vs. 53%) or severe aortic stenosis (4% vs. 18%) (Figure 2). Consequently, SARS-COV-2-positive patients less commonly underwent coronary angiography (with or without intervention) or aortic valve replacement (TAVI or surgery). For the subgroup of patients presenting with STEMI, Supplementary Table 1 shows the time from symptom onset to hospital arrival, arrival at the catheterization laboratory, and balloon dilatation in relation to SARS-COV-2 positivity. Moreover, patients with SARS-COV-2 more commonly had fever at presentation (29% vs. 5%), and more commonly required admission to ICU (49% vs. 13%) (Table 1).

Laboratory analyses at time point 1 (closest to admission) showed higher levels of hs-CRP in cases vs. controls (93.5 ± 89.3 vs. 26.7 ± 54.0; *p* < 0.001), without differences in leucocytes. No differences were found for cardiac biomarkers (CK-MB, hs-TnT). At time point 2 (closest to discharge), hs-CRP and leukocytes were higher and hemoglobin was lower in cases, whereas troponin levels were higher in controls (Table 2). Focusing on hs-TnT, in a subanalysis excluding patients with ACS we found no significant differences in relation to SARS-COV-2 positivity at either of the two time points.

**Table 2:** Laboratory analyses during index hospitalization.

	All patients *N* = 538	Controls *N* = 416	Cases *N* = 122	Difference (95% CI)	*p* value
Timepoint 1^a^					
Leucocytes (G/L)	*n* = 525, 9.6 ± 4.7	*n* = 405, 9.6 ± 4.2	*n* = 120, 9.5 ± 6.1	0.1 (−0.8; 1.1)	0.77
Hemoglobin (g/L)	*n* = 525, 132.6 ± 23.1	*n* = 405, 133.8 ± 22.5	*n* = 120, 128.7 ± 24.7	5.1 (0.4; 9.8)	0.03
Platelets (G/L)	*n* = 524, 226.7 ± 78.5	*n* = 405, 229.7 ± 78.5	*n* = 119, 216.4 ± 78.1	13.2 (−2.8; 29.3)	0.11
hs-CRP (mg/L)	*n* = 373, 44.1 ± 71.3	*n* = 276, 26.7 ± 54.0	*n* = 97, 93.5 ± 89.3	−66.8 (−81.9; −51.8)	<0.001
CK (U/L)	*n* = 499, 377.7 ± 834.6	*n* = 397, 410.1 ± 905.9	*n* = 102, 251.3 ± 443.5	158.8 (−22.9; 340.4)	0.09
CKMB (*μ*g/L)	*n* = 205, 65.5 ± 112.9	*n* = 142, 64.5 ± 104.2	*n* = 63, 67.6 ± 131.3	−3.1 (−36.7; 30.6)	0.86
hs-TnT (ng/L)	*n* = 500, 774.2 ± 2,556.7	*n* = 394, 859.1 ± 2,697.2	*n* = 106, 458.7 ± 1,925.8	400.4 (−148.5; 949.4)	0.15
Excluding patients with ACS	*n* = 252, 178.7 ± 1,163.3	*n* = 178, 211.9 ± 1,380.1	*n* = 74, 98.8 ± 160.4	113.1 (−203.3; 429.5)	0.48
eGFR (ml/min)	*n* = 513, 68.2 ± 25.8	*n* = 404, 69.3 ± 24.8	*n* = 109, 64.2 ± 29.1	5.1 (−0.4; 10.5)	0.07
Timepoint 2^b^					
Leucocytes (G/L)	*n* = 446, 8.5 ± 3.7	*n* = 335, 8.2 ± 2.9	*n* = 111, 9.2 ± 5.3	−1.0 (−1.8; −0.2)	0.01
Hemoglobin (g/L)	*n* = 447, 120.2 ± 23.3	*n* = 335, 122.5 ± 22.3	*n* = 112, 113.3 ± 25.0	9.2 (4.2; 14.1)	<0.001
Platelets (G/L)	*n* = 444, 229.3 ± 101.4	*n* = 334, 221.4 ± 99.1	*n* = 110, 253.2 ± 104.9	−31.8 (−53.5; −10.1)	0.004
hs-CRP (mg/L)	*n* = 244, 38.9 ± 56.6	*n* = 159, 29.5 ± 37.5	*n* = 85, 56.3 ± 78.4	−26.7 (−41.3; −12.1)	<0.001
CK (U/L)	*n* = 416, 710.9 ± 2,110.6	*n* = 340, 672.1 ± 1,220.9	*n* = 76, 884.5 ± 4,227.6	−212.4 (−738.7; 313.8)	0.43
CKMB (μg/L)	*n* = 241, 56.4 ± 107.2	*n* = 178, 63.4 ± 114.7	*n* = 63, 36.6 ± 80.0	26.8 (−3.9; 57.5)	0.09
hs-TnT (ng/L)	*n* = 418, 1,443.9 ± 3,301.8	*n* = 338, 1,647.2 ± 3,512.4	*n* = 80, 585.1 ± 1,998.3	1,062.1 (261.2; 1,863.0)	0.01
Excluding patients with ACS	*n* = 196, 142.3 ± 324.7	*n* = 138, 144.7 ± 362.9	*n* = 58, 136.8 ± 209.9	7.8 (−92.3; 107.9)	0.88
eGFR (ml/min)	*n* = 473, 66.9 ± 27.6	*n *= 377, 67.8 ± 26.5	*n* = 96, 63.7 ± 31.7	4.0 (−2.2; 10.2)	0.20

CK, creatine kinase; eGFR, estimated glomerular filtration rate; hs-CRP, high-sensitivity C-reactive protein; hs-TnT, high-sensitivity troponin T.

^a^ Time point closest to admission for index hospitalization.

^b^ Time point closest to hospital discharge.

### In-hospital, 30-day, and 1-year outcomes

**Figure 3:**
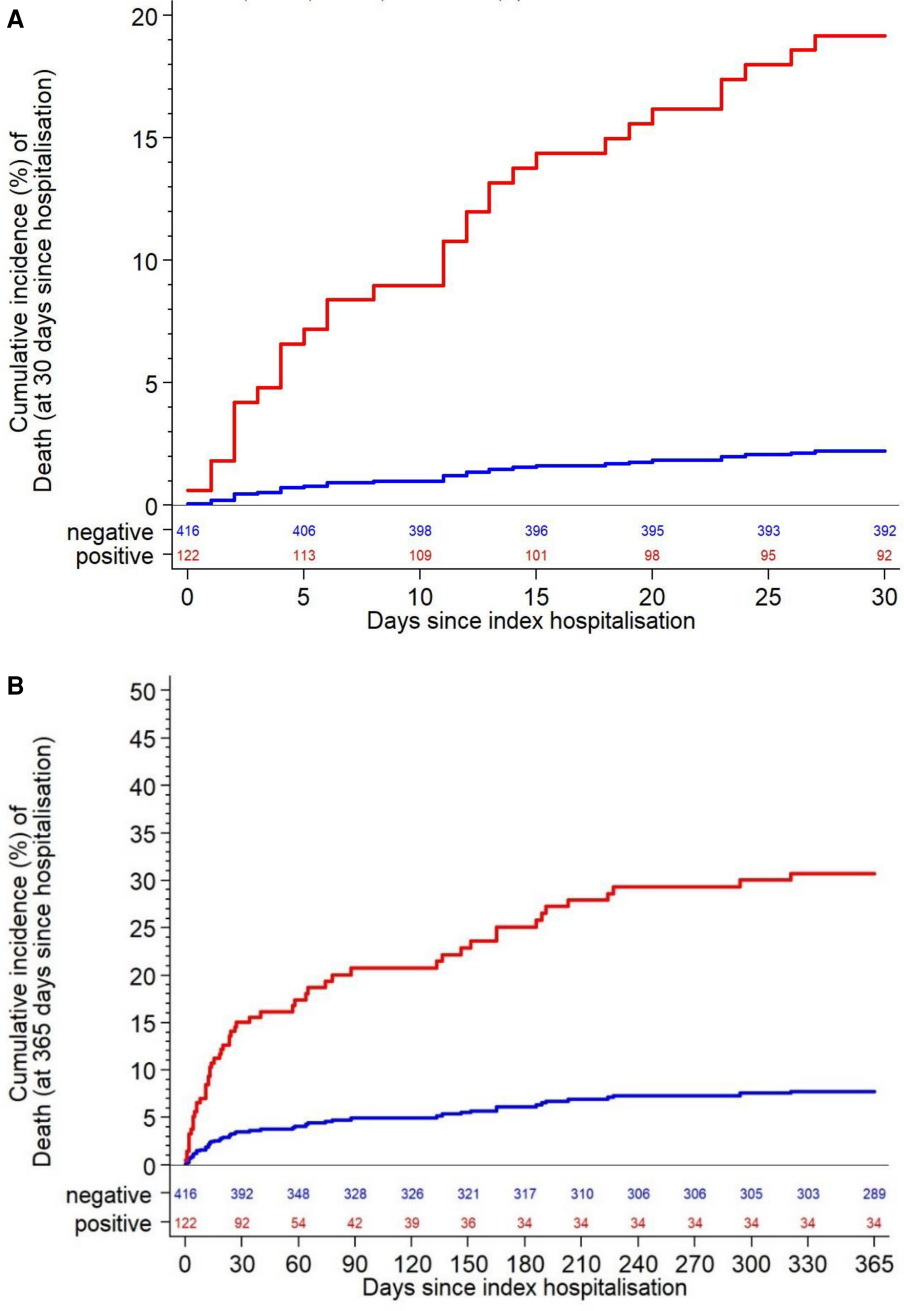
All-cause death within 30 days (**A**) and within 1 year (**B**).

Table 3 summarizes in-hospital outcomes. More SARS-COV-2-positive patients died in hospital (16% vs. 1%; *p* < 0.001). This was driven by significant differences in both cardiovascular death (3% vs. 0.5%; *p* = 0.03) and non-cardiovascular death (12% vs. 1%; *p* < 0.001). Acute kidney injury was more common in cases vs. controls (8% vs. 2%; *p* < 0.001). At 30 days, higher mortality was seen in cases vs. controls [19.2% vs. 2.2%, HR 9.45 (4.37–20.44); *p* < 0.001] (Figure 3), driven primarily by higher non-cardiovascular mortality (14.1% vs. 0.7%; *p* < 0.001) and to a lesser extent by higher cardiovascular mortality (4.1% vs. 0.7%; *p* = 0.02). Acute kidney injury, major arrhythmia, and hospitalization for heart failure within 30 days were all significantly more frequent in patients with SARS-COV-2 infection (Table 4).

**Table 3:** In-hospital clinical outcomes.

	Controls *N* = 416	Cases *N* = 122	Difference (95% CI)	*p*-value
Death	5 (1%)	20 (16%)	−15% (−19%; −11%)	<0.001
Cardiovascular death	2 (0%)	4 (3%)	−3% (−5%; −1%)	0.03
Non-cardiovascular death	3 (1%)	15 (12%)	−12% (−15%; −8%)	<0.001
Unclear death	0 (0%)	1 (1%)	−1% (−2%; 0%)	0.23
Myocardial infarction	3 (1%)	3 (2%)	−2% (−4%; 0%)	0.13
Unplanned revascularisation	0 (0%)	0 (0%)		
Cerebrovascular event	7 (2%)	1 (1%)	1% (−2%; 3%)	0.69
Stroke (ischemic or hemorhagic)	4 (1%)	1 (1%)	0% (.%;.%)	1.000
Acute kidney injury	7 (2%)	10 (8%)	−7% (−10%; −3%)	0.001
Major arrhythmia	25 (6%)	18 (15%)	−9% (−14%; −3%)	0.004

Depicted are counts (%).

**Table 4:** Clinical outcomes at 30 days and one year^a^.

	Outcomes at 30 days				Outcomes at 1 year			
	Controls *N* = 428	Cases *N* = 132	Subdistribution hazard ratio (95% CI)	*p*-value	Controls *N* = 428	Cases *N* = 132	Subdistribution hazard ratio (95% CI)	*p*-value
Death	9 (2.2%) [0]	23 (19.2%) [0]	9.45 (4.37–20.44)	<0.001	29 (7.7%) [0]	26 (30.7%) [0]	4.30 (2.58–7.17)	<0.001
Cardiovascular death	3 (0.7%) [6]	5 (4.1%) [18]	5.76 (1.38–24.07)	0.02	9 (2.4%) [20]	5 (5.5%) [21]	2.28 (0.80–6.50)	0.12
Non-cardiovascular death	3 (0.7%) [6]	17 (14.3%) [6]	20.64 (6.05–70.40)	<0.001	13 (3.5%) [16]	19 (21.8%) [7]	6.62 (3.42–12.82)	<0.001
Unclear death	3 (0.7%) [6]	1 (0.8%) [22]	1.14 (0.12–10.87)	0.91	7 (1.9%) [22]	2 (2.5%) [24]	1.22 (0.26–5.79)	0.81
Myocardial infarction	3 (0.7%) [8]	3 (2.5%) [23]	3.44 (0.70–16.96)	0.13	7 (1.8%) [27]	3 (3.2%) [26]	1.73 (0.47–6.37)	0.41
Unplanned revascularisation	0 (0.0%) [9]	0 (0.0%) [23]			0 (0.0%) [29]	1 (1.7%) [26]		
Cerebrovascular event	7 (1.7%) [9]	1 (0.8%) [23]	0.49 (0.06–3.99)	0.51	11 (2.9%) [26]	2 (2.2%) [26]	0.72 (0.16–3.31)	0.67
Stroke (ischemic or hemorrhagic)	4 (1.0%) [9]	1 (0.8%) [23]	0.85 (0.10–7.67)	0.89	6 (1.6%) [27]	2 (2.2%) [26]	1.33 (0.26–6.80)	0.73
Acute kidney injury	8 (1.9%) [8]	9 (7.5%) [19]	3.96 (1.54–10.24)	0.004	9 (2.3%) [27]	12 (11.1%) [22]	5.04 (2.11–12.09)	<0.001
Major arrhythmia	26 (6.2%) [8]	18 (14.8%) [18]	2.47 (1.36–4.48)	0.003	32 (8.0%) [27]	22 (20.3%) [21]	2.66 (1.55–4.58)	<0.001
Hospitalization for heart failure	3 (0.7%) [9]	4 (3.4%) [23]	4.65 (1.03–20.54)	0.04	18 (5.0%) [27]	5 (6.6%) [26]	1.27 (0.48–3.32)	0.63

Depicted are counts with the Aalen-Johanssen estimator of the cumulative incidence (%) [in square brackets the number of patients with the competing event occurring before the event of interest]. Subdistribution ratio of the hazards using the Fine & Grey methodology with 95% confidence interval (CI) and *p*-value comparing controls vs. cases.

^a^ First events of each event type under competing risk with death.

At one-year follow-up, all-cause mortality was higher in SARS-COV-2-positive cases vs. controls [28.7% vs. 7.6%, HR 4.30 (2.58–7.17); *p* < 0.001] (Figure 3). This was driven by higher non-cardiovascular mortality [20.4% vs. 3.4%, HR 6.62 (3.42–12.82), *p* < 0.001], without a significant difference in cardiovascular mortality [5.3% vs. 2.3%, HR 2.28 (0.80–6.50); *p* = 0.12]. Acute kidney injury and major arrhythmia were also significantly higher in cases vs. controls (10.8% vs. 2.3% and 19.8% vs. 8.0%, respectively; *p* < 0.001 for both) (Table 4).

### Outcomes in the propensity score-matched cohort

Because of the imbalance in clinical presentation and baseline characteristics between SARS-COV-2-positive and -negative patients, we performed propensity score matching in a 1:1 fashion based on selected baseline variables and compared outcomes between matched cases (*n* = 93) and controls (*n* = 93). The 2 groups were well balanced at baseline (Supplementary Table 2), with the exception of more frequent presentation with NYHA Class IV in patients with SARS-COV-2 infection. In-hospital mortality was higher in cases vs. controls (15% vs. 4%, *p* = 0.02), driven by higher non-cardiovascular mortality (11% vs. 2%, *p* = 0.03), without a difference in cardiovascular mortality (3% vs. 2%) (Table 5). Similar differences were confirmed with respect to 30-day outcomes, indicating greater all-cause mortality (18.3% vs. 5.5%, *p* = 0.01) and non-cardiovascular mortality (13.1% vs. 2.2%, *p* = 0.02) without significant differences in cardiovascular death. Along the same lines, all-cause and non-cardiovascular mortality were higher at 1 year in cases vs. controls (24.1% vs. 10.6%, *p* = 0.02 and 16.1% vs. 4.7%, *p* = 0.02, respectively), without a significant difference in cardiovascular mortality (4.3% vs. 2.2%; *p* = 0.42). There were no differences in myocardial infarction, unplanned revascularization, cerebrovascular events, or hospitalization for heart failure at any time point during follow-up (Table 6, Supplementary Figure 1).

**Table 5:** In-hospital clinical outcomes in the matched cohort (*n* = 186).

	Controls *N* = 93	Cases *N* = 93	Difference (95% CI)	*p*-value
Death	4 (4%)	14 (15%)	−11% (−19%; −2%)	0.02
Cardiovascular death	2 (2%)	3 (3%)	−1% (−6%; 4%)	1.00
Non-cardiovascular death	2 (2%)	10 (11%)	−9% (−16%; −2%)	0.03
Unclear death	0 (0%)	1 (1%)	−1% (−3%; 1%)	1.00
Myocardial infarction	1 (1%)	2 (2%)	−1% (−5%; 3%)	1.00
Unplanned revascularisation	0 (0%)	0 (0%)		
Cerebrovascular event	0 (0%)	0 (0%)		
Stroke (ischemic or hemorrhagic)	0 (0%)	0 (0%)		
Acute kidney injury	3 (3%)	6 (6%)	−3% (−9%; 3%)	0.50
Major arrhythmia	9 (10%)	10 (11%)	−1% (−10%; 8%)	1.00

**Table 6:** Clinical outcomes at 30 days and 1 year in the matched cohort (*n* = 186)^a^.

	Outcomes at 30 days				Outcomes at 1 year			
	Negative *N* = 93	Positive *N* = 93	Subdistribution hazard ratio (95% CI)	*P* value	Negative *N* = 93	Positive *N* = 93	Subdistribution hazard ratio (95% CI)	*p*-value
Death	5 (5.6%) [0]	17 (18.6%) [0]	3.55 (1.31–9.62)	0.01	9 (10.8%) [0]	19 (25.7%) [0]	2.46 (1.13–5.33)	0.02
Cardiovascular death	2 (2.2%) [3]	4 (4.4%) [13]	2.02 (0.37–10.96)	0.42	2 (2.2%) [7]	4 (4.4%) [15]	2.02 (0.37–10.96)	0.42
Non-cardiovascular death	2 (2.2%) [3]	12 (13.3%) [5]	6.21 (1.40–27.65)	0.02	4 (4.8%) [5]	13 (17.1%) [6]	3.66 (1.24–10.81)	0.02
Unclear death	1 (1.1%) [4]	1 (1.1%) [16]	0.98 (0.06–15.17)	0.99	3 (4.0%) [6]	2 (3.6%) [17]	0.80 (0.13–4.77)	0.81
Myocardial infarction	1 (1.1%) [5]	2 (2.2%) [17]	2.01 (0.18–21.89)	0.57	2 (2.5%) [9]	2 (2.8%) [19]	1.09 (0.16–7.18)	0.93
Unplanned revascularisation	0 (0.0%) [5]	0 (0.0%) [17]			0 (0.0%) [9]	0 (0.0%) [19]		
Cerebrovascular event	0 (0.0%) [5]	0 (0.0%) [17]			1 (1.6%) [9]	1 (2.4%) [19]	1.31 (0.08–21.05)	0.85
Stroke (ischemic or hemorrhagic)	0 (0.0%) [5]	0 (0.0%) [17]			0 (0.0%) [9]	1 (2.4%) [19]		
Acute kidney injury	3 (3.3%) [5]	5 (5.4%) [14]	1.69 (0.40–7.06)	0.47	3 (3.5%) [9]	7 (8.5%) [16]	2.48 (0.63–9.80)	0.19
Major arrhythmia	9 (9.6%) [4]	10 (11.0%) [13]	1.14 (0.47–2.78)	0.78	10 (11.7%) [7]	13 (16.6%) [15]	1.42 (0.62–3.25)	0.41
Hospitalization for heart failure	1 (1.1%) [5]	3 (3.4%) [17]	2.96 (0.31–28.48)	0.35	7 (9.8%) [9]	4 (7.3%) [19]	0.69 (0.21–2.25)	0.54

^a^ First event of each event type under competing risk with death.

### Exploratory analyses

Because of the chronic nature of severe aortic stenosis as compared with the other clinical diagnoses of patients included in this cohort, we performed *post hoc* exploratory analyses excluding patients with severe aortic stenosis (*n* = 79). These analyses, detailed in Supplementary Tables 3–6, showed essentially similar results with respect to baseline characteristics and laboratory findings as in the main analyses in the entire patient cohort of 538 patients. With respect to clinical outcomes, we found overall fewer cerebrovascular and major arrhythmia events throughout the duration of follow-up than in the entire cohort; clinical outcomes in cases vs. controls showed similar differences as in the entire cohort, including a higher all-cause and non-cardiovascular mortality among SARS-COV-2-positive patients at one year.

Prompted by the high prevalence of diabetes mellitus among SARS-COV-2-positive patients, we performed additional exploratory analyses of baseline characteristics, laboratory findings and clinical outcomes in cases stratified by diabetic status (Supplementary Tables 7–10). These analyses showed that SARS-COV-2-positive patients with, as compared to those without diabetes, were older, had lower hemoglobin and eGFR levels at baseline, and more frequently developed acute kidney injury within one year, whereas other clinical outcomes showed no statistically significant differences.

Finally, in order to explore characteristics of SARS-COV-2-positive patients with marked clinical deterioration, we compared baseline clinical and laboratory findings between SARS-COV-2-positive patients who were admitted to an ICU during index hospitalization (*n* = 60) vs. those who were not (*n* = 62). We found that patients transferred to ICU tended to be older, presented with more advanced dyspnea, and had higher leukocyte counts and hs-CRP levels already at the first time point of blood sampling (Supplementary Table 11 and 12).

## Discussion

This observational registry study compared clinical outcomes in patients who were hospitalized primarily due to an acute cardiac condition in relation to the presence or absence of SARS-COV-2 infection during index hospitalization in 5 Swiss Cardiology Departments. We found that SARS-COV-2 infection portended a markedly worse prognosis starting at the in-hospital period and extending up to one year of follow-up. Mortality was more than 9-fold higher at 30 days and more than 4-fold higher at one year in patients who were SARS-COV-2-positive at the time of hospital admission. Notably, the difference was driven by an excess in non-cardiac rather than cardiac mortality throughout the duration of patient follow-up.

Several lines of evidence have indicated that infection with the SARS-COV-2 virus can exert adverse cardiac effects. First, myocardial damage as expressed by the increase of myocardial biomarkers has been shown in COVID-19 (6). Furthermore, magnetic resonance imaging has shown evidence of myocardial edema and inflammation in patients recovering from COVID-19 without clinically evident cardiac manifestations (16, 17). Of prognostic relevance, documentation of acute cardiac injury among patients with respiratory infection as a primary clinical manifestation of COVID-19 has consistently been shown to adversely affect survival (5, 6). Further research has suggested that SARS-COV-2 infection can affect the cardiovascular system via different mechanisms including a pro-arrhythmogenic and prothrombotic potential (1, 2, 11, 18). In addition, growing evidence has clearly shown that pre-existing CVD (7–9) as well as classical CVD risk factors (19) have a strong negative impact on patient prognosis and survival. Thereby, accumulating experience from almost 3 years of the pandemic has highlighted multiple systemic manifestations of COVID-19 that can profoundly affect the cardiovascular system.

Our study, focusing on patients who were hospitalized for a wide range of acute cardiac conditions in Swiss Cardiology Departments, found excess in-hospital mortality among SARS-COV-2-positive patients. A nationwide study from Spain focusing only on patients presenting with STEMI reported greater in-hospital mortality among 91 SARS-COV-2-positive patients compared with 919 controls (20). In contrast, the present study included a broad range of acute cardiac conditions and followed patients up to one year after the index hospitalization. The finding that the substantially higher mortality in SARS-COV-2-positive patients was attributed mainly to non-cardiovascular deaths, particularly during the later (after 30 days) stage of follow-up likely relates to the systemic nature of COVID-19 and the potential for longer-term sequelae affecting different organs and systems. The one-year all-cause mortality rate in the control (SARS-COV-2-negative) group of our study appears to be in line with our prior, pre-pandemic experience in ACS patients (21) as well as reports of real-world studies (outside the COVID-19 pandemic) including patients with ACS (22, 23) or patients undergoing TAVI (24). Nonetheless, as noted in previous relevant reports (7, 25, 26), cardiovascular outcomes might have been adversely affected—also unrelated to presence of absence of SARS-COV-2 infection *per se*—by confounding factors such as delayed referral or patients’ fear to access hospitals during COVID-19 surges.

Our finding of significantly more frequent kidney injury among SARS-COV-2-positive cases vs. controls, coupled with the reported negative impact of renal function deterioration on post-COVID-19 outcomes (27), might explain at least in part the marked non-cardiovascular mortality difference between SARS-COV-2-positive cases and controls in our study. Although the mechanisms underlying these findings remain largely unclear, they highlight the excessive risk for patients with concomitant acute cardiac conditions and SARS-COV-2 infection, and point to the need for multifaceted, holistic management with prolonged and close surveillance of these patients. Treatment in this very high-risk setting should therefore focus on guideline-directed therapies for the acute cardiac problem without unjustified delays (28), as well as appropriate measures including dedicated antiviral drugs (29) and vaccination to boost long-term immunity where indicated (30).

Previous studies have reported delays in performance of primary PCI for SARS-COV-2-positive STEMI patients (20, 31), longer ischemic times for STEMI patients in the pandemic vs. the pre-pandemic era (32, 33), and longer time between initial diagnosis of severe aortic stenosis and performance of TAVI during the COVID-19 pandemic surges as compared with pre-pandemic conditions (34). These findings have been interpreted as both patient-related delays (apparently due to fear of many patients to access hospitals during pandemic surges (28, 35, 36) and system-related delays (25), and have been associated with worse outcomes. In the present registry, time from symptom onset to hospital admission and intervention were captured for the subgroup of patients with STEMI. Although differences were statistically not significant (possibly due to small numbers of COVID-positive STEMI cases), numerically we did observe longer times for SARS-VOV-2-positive vs. negative patients (Supplementary Table 1). Thereby, and although strong conclusions on causality cannot be conferred, we cannot exclude that delays in interventional treatment related to SARS-COV-2 positivity and generally compared with pre-pandemic standards affected the reported differences in clinical outcomes.

This study included fewer patients with than without SARS-COV-2 infection. This may relate to the pragmatic study design limited to the evaluation of patients admitted to Cardiology rather than other services (e.g., internal medicine or pulmonology clinics for patients with primarily respiratory COVID-19 manifestations and concomitant cardiac conditions), and the relatively low incidence of SARS-COV-2 positivity in the given clinical setting at the time the study took place. The number of SARS-COV-2 positive patients in our study is modest but comparable to previous reports assessing differences in patients with cardiac conditions in relation to SARS-CO-2- positivity (7, 20, 37, 38). As in all observational studies including registries during the COVID-19 pandemic (20, 25, 31, 37), the number of patients enrolled was not based on an *a priori* power analysis and sample size calculation. However, and notwithstanding the inherent limitations common to observational studies prohibiting direct causality inferences, the markedly higher mortality in SARS-COV-2-positive patients appears to be plausible in view of cumulating evidence in the field (1, 2, 26), and reflects a clinically relevant difference in prognosis in this context.

While COVID-19 has been suggested to increase the risk of myocardial infarction and other thrombotic complications (26, 39), in our study the proportion of patients presenting with ACS was two-fold lower for SARS-COV-2-negative vs. positive patients. This might relate to the previously reported observation of a decline in AMI admissions during the pandemic (28, 35, 36); moreover, unmeasured bias in the selection of controls cannot be definitively excluded in this observational registry. Imbalances in clinical presentation as well as baseline characteristics between SARS-COV-2-positive cases and controls were inevitable in this context, in line with previous case-control studies assessing the impact of COVID-19 among patients with primarily cardiac diseases (7, 20). Because clinical prognosis is very likely to vary substantially across the heterogeneous spectrum of primary cardiac diagnoses in our cohort (ranging from new-onset atrial fibrillation to ACS to older, polymorbid patients with severe aortic stenosis), and in view of imbalanced risk factors such as diabetes mellitus [which has been shown to affect COVID-19 outcomes (19)], we performed propensity score matching aiming to address inherent biases and confounders. Importantly, the main findings concerning mortality in the entire study population held true for the matched cohort with comparable cardiac diagnoses and baseline characteristics in patients with vs. without SARS-COV-2 infection.

Laboratory analyses showed that hs-CRP was higher in patients with SARS-COV-2 infection. Although in the context of ACS—i.e., the main cardiac diagnosis in almost half of all patients in this study—an inflammatory response is known to result in elevated inflammatory biomarkers, concomitant SARS-COV-2 infection did lead to much greater CRP elevations compared with SARS-COV-2-negative patients. Indeed, inflammation, and in particular the NLRP3-interleukin pathway is known to affect outcomes in both ACS and COVID-19. It is also notable that higher hs-CRP levels and leukocytes counts at baseline, along with more advanced dyspnea at initial presentation, were seen in those SARS-COV-2-positive patients who eventually required ICU transfer during index hospitalization. As it relates to the increase in troponin levels, similar to previous reports, in our cohort it was not possible to distinguish the potential contribution of ischemic damage in the case of ACS vs. infection-induced myocardial injury. The differential diagnosis between ACS and COVID-19-induced myocardial necrosis, and the differentiation in cases of coexistence of both entities, has posed challenges in view of overlap in presenting (cardiac or respiratory) symptoms as well as biomarker elevations (7, 18, 26, 40). Our findings of higher peak levels in controls as compared with SARS-COV-2-positive cases are likely confounded by the 2-fold greater proportion of ACS in SARS-COV-2-negative vs. positive patients. Indeed, our subanalysis excluding patients with ACS showed no significant differences in relation to SARS-COV-2 positivity.

This study has several limitations. First, our cohort mainly included patients enrolled during the first and second SARS-COV-2 pandemic waves in Switzerland, and our findings cannot be extrapolated to later stages of the pandemic course due to differences in population-level immunity (either infection-induced or following vaccination) and the availability of treatments specific to SARS-COV-2. Second, because the study was designed at the very early stages of the pandemic in March 2020 (with no vaccination available at that time), vaccination status was not recorded in the dataset for patients enrolled at later stages of the study. Third, while we intentionally included patients with a broad range of acute cardiac conditions, the heterogeneity in initial cardiac diagnoses inevitably confounded subsequent clinical outcomes. Although the propensity score-matched cohort sought to address this limitation, unmeasured confounding factors may still exist. Fourth, pulmonary testing (e.g., specific imaging or blood gas analyses) was performed as clinically indicated in patients with signs of pneumonia or worsening respiratory function, but these data were not systematically collected in the present registry. Previous studies have addressed the impact of in-hospital respiratory impairment on clinical outcomes in patients with acute cardiac disease and COVID-19 (41).

In conclusion, in this observational study of patients hospitalized for a broad range of acute cardiac diseases, SARS-COV-2 infection at index hospitalization was associated with a markedly increased all-cause and non-cardiovascular mortality throughout the one-year follow-up. These findings reinforce the need for effective, multifaceted management of both cardiac and non-cardiac morbidities, and prolonged surveillance in patients with acute cardiac conditions complicated by SARS-COV-2 infection.

## Data Availability

The raw data supporting the conclusions of this article will be made available by the authors, without undue reservation.
