# Supplementary material for: Effects of SARS-COV-2 infection on outcomes in patients hospitalized for acute cardiac conditions. A prospective, multicenter cohort study (Swiss Cardiovascular SARS-CoV-2 Consortium)
Source: Front Cardiovasc Med. 2023 Oct 13;10:1203427. doi: 10.3389/fcvm.2023.1203427 (PMC10613056; doi:10.3389/fcvm.2023.1203427)
Supplement: Supplementary file 1 [file Datasheet1.pdf]

Supplementary Material

Effects of SARS-COV-2 infection on outcomes in patients hospitalized for acute cardiac conditions. A prospective, multicenter cohort study

Swiss Cardiovascular SARS-CoV-2 Consortium

Konstantinos C Koskinas[1], Raphael Twerenbold[2,3,4], David Carballo[5], Christian M Matter[6], Stephane Cook[7], Dik Heg[8], Andre Frenk[1], Stephan Windecker[1], Stefan Osswald[2], Thomas F Lüscher[9,10], Francois Mach[5]

[1]Bern University Hospital Inselspital, Bern, Switzerland

[2]Basel University Hospital, Basel, Switzerland

[3]University Center of Cardiovascular Science & Department of Cardiology, University Heart and Vascular Center Hamburg, University Medical Center Hamburg-Eppendorf, Hamburg, Germany

[4]German Center for Cardiovascular Research (DZHK) Partner Site Hamburg–Kiel–Lübeck, Germany

[5]Geneva University Hospitals, Geneva, Switzerland

[6]Zurich University Hospital, Zurich, Switzerland

[7]Fribourg Canton Hospital, Fribourg, Switzerland

[8]CTU Bern, University of Bern, Bern, Switzerland.

[9]Royal Brompton & Harefield Hospitals and National Heart and Lung Institute, Imperial College, London, UK.

[10]Center for Molecular Cardiology, University of Zurich, Zurich, Switzerland.

*** Correspondence:**
Konstantinos Koskinas, MD

Department of Cardiology

Bern University Hospital – Inselspital

Freiburgstrasse 18

CH-3010 Bern

Mail: konstantinos.koskinas@insel.ch

Supplemental Figures

Supplemental Figure 1. All-cause death within 30 days (**A**) and within 1 year (**B**) in the matched cohort (n=186)

A

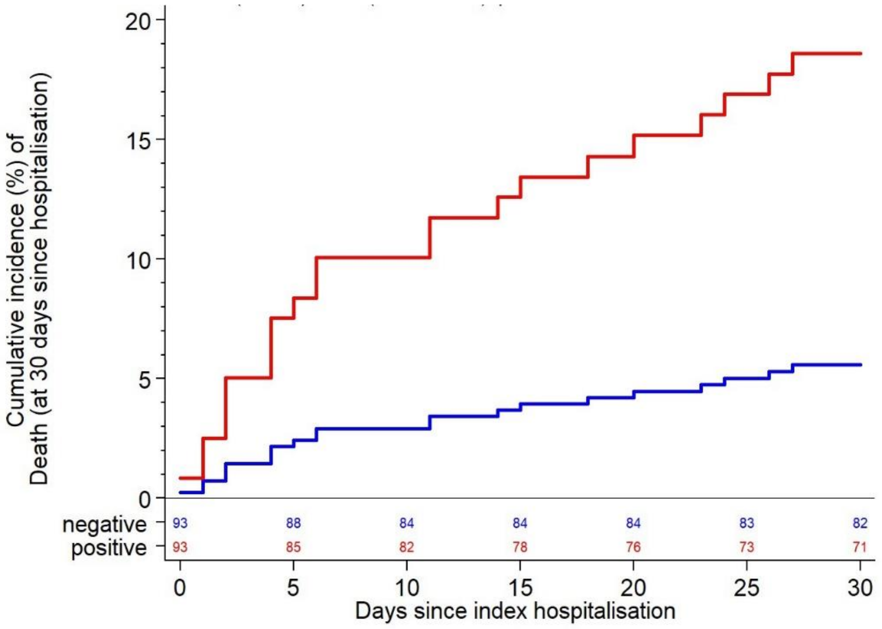

B

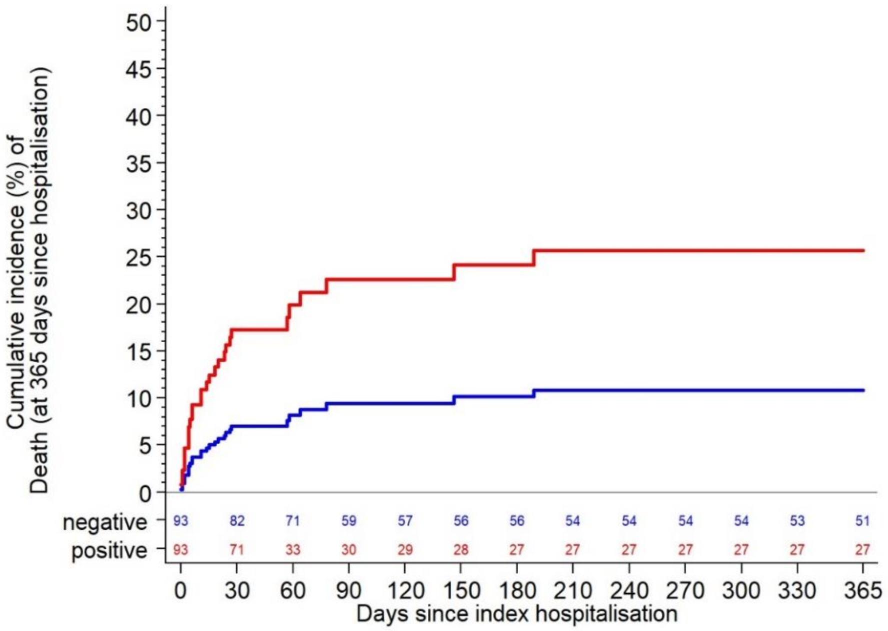

Supplemental Tables

Supplemental Table 1. Time interval between symptom onset, hospital arrival, and coronary intervention in the subgroup of patient with STEMI

	All patients with STEMI N=103	Controls N=95	Cases N=8	p Value
Time of symptom onset ≤ 24 hours	82 of 103 (80%)	76 of 95 (80%)	6 of 8 (75%)	0.66
Symptom onset to hospital arrival time (min)	166.0 (70.0; 576.0)	160.0 (71.8; 556.2)	245.0 (80.0; 1043.2)	0.43
Symptom onset to catheter lab time (min)	189.0 (103.5; 624.5)	180.0 (104.0; 647.0)	292.0 (69.0; 1389.0)	0.91
Symptom onset to balloon/stenting time (min)	209.0 (122.0; 672.2)	201.5 (122.2; 656.0)	356.5 (122.0; 1509.8)	0.42
Time of symptom onset >24 hours	21 of 103 (20%)	19 of 95 (20%)	2 of 8 (25%)	0.66
>24-48 hours	12 (57%)	11 (58%)	1 (50%)	1.00
>48-72 hours	3 (14%)	3 (16%)	0 (0%)	1.00
>72 hours - 7 days	2 (10%)	2 (11%)	0 (0%)	1.00
>7 days	4 (19%)	3 (16%)	1 (50%)	0.35

Depicted are medians (25%; 75% quartiles) and counts with percentages of patients with non-missing data (%). P-values from t-tests, Mann-Whitney U-tests or Fisher's tests (or chi square tests in case more than 2 x 2 comparisons).

Supplemental Table 2. **Baseline patient characteristics in the matched cohort (n=186)**

	All patients N=186	Controls n=93	Cases N=93	Difference (95% CI)	p Value
Age (years)	67.9 ± 15.0	68.6 ± 14.7	67.3 ± 15.3	1.3 (-3.0; 5.6)	0.555
Male gender	145 (78%)	70 (75%)	75 (81%)	-5% (-17%; 7%)	0.480
Diagnosis at index hospitalizazion					0.987
Acute coronary syndrome	61 (33%)	29 (31%)	32 (34%)	-3% (-17%; 0%)	0.755
New-onset major arrhythmia	50 (27%)	25 (27%)	25 (27%)	0%	1.000
Acute heart failure	58 (31%)	31 (33%)	27 (29%)	4% (-9%; 18%)	0.635
Severe symptomatic aortic stenosis	10 (5%)	5 (5%)	5 (5%)	0%	1.000
Hospitalization related to adult congenital heart disease	2 (1%)	1 (1%)	1 (1%)	0%	1.000
Clinically suspected myocarditis	5 (3%)	2 (2%)	3 (3%)	-1% (-6%; 4%)	1.000
Diabetes mellitus	73 (39%)	37 (40%)	36 (39%)	1% (-13%; 15%)	1.0
Current smoker	78 (42%)	35 (38%)	43 (46%)	-9% (-23%; 6%)	0.298
Hypercholesterolemia	86 (46%)	41 (44%)	45 (48%)	-4% (-19%; 10%)	0.659
Previous myocardial infarction	48 (26%)	27 (29%)	21 (23%)	6% (-6%; 19%)	0.402
History of malignancy	28 (15%)	19 (20%)	9 (10%)	11% (0%; 21%)	0.064
Chronic obstructive lung disease	18 (10%)	10 (11%)	8 (9%)	2% (-6%; 11%)	0.805
Clinically relevant valvular disease	18 (10%)	10 (11%)	8 (9%)	2% (-6%; 11%)	0.805
Type of aortic valvular disease	n = 16	n = 10	n = 6		0.726
Severe native aortic valve stenosis	10 (63%)	7 (70%)	3 (50%)	20% (-32%; 72%)	0.607
Degenerated aortic bioprosthesis	2 (13%)	1 (10%)	1 (17%)	-7% (-43%; 29%)	1.000
Severe aortic regurgitation	4 (25%)	2 (20%)	2 (33%)	-13% (-60%; 33%)	0.604
NYHA class	n = 175	n = 92	n = 83		0.006
NYHA I	59 (34%)	40 (43%)	19 (23%)	21% (7%; 34%)	0.006
NYHA II	32 (18%)	15 (16%)	17 (20%)	-4% (-16%; 7%)	0.558
NYHA III	35 (20%)	20 (22%)	15 (18%)	4% (-8%; 16%)	0.575
NYHA IV	49 (28%)	17 (18%)	32 (39%)	-20% (-33%; -7%)	0.004
Cardiogenic shock (Killip 4)	18 (10%)	8 (9%)	10 (11%)	-2% (-11%; 6%)	0.805
Fever	33 (18%)	13 (14%)	20 (22%)	-8% (-20%; 3%)	0.178

Intervention performed during index hospitalization					
Coronary procedure	54 (29%)	33 (35%)	21 (23%)	13% (-0%; 26%)	0.075
TAVI	7 (4%)	5 (5%)	2 (2%)	3% (-2%; 9%)	0.444
Intervention for major arrhythmia	23 (12%)	13 (14%)	10 (11%)	3% (-6%; 13%)	0.657
Patient transfer to Intensive Care Unit	65 (35%)	33 (35%)	32 (34%)	1% (-13%; 15%)	1.000

Supplemental Table 3. Baseline characteristics of enrolled patients excluding patients with severe aortic stenosis

	All patients<br>N = 459	Controls<br>N = 342	Cases<br>N = 117	Difference (95% CI)	p Value
Age (years)	66.2 ± 14.9	65.7 ± 14.9	67.6 ± 14.8	-1.8 (-5.0; 1.3)	0.25
Male sex	352 (77%)	264 (77%)	88 (75%)	2% (-7%; 11%)	0.70
Diagnosis at index hospitalization					<0.001
Acute coronary syndrome	254 (55%)	222 (65%)	32 (27%)	38% (28%; 47%)	<0.001
New-onset major arrhythmia	75 (16%)	37 (11%)	38 (32%)	-22% (-29%; -14%)	<0.001
Acute heart failure	115 (25%)	72 (21%)	43 (37%)	-16% (-25%; -7%)	0.001
Hospitalization related to adult congenital heart disease	4 (1%)	3 (1%)	1 (1%)	0%	1.00
Clinically suspected myocarditis	11 (2%)	8 (2%)	3 (3%)	0% (-3%; 3%)	1.00
Diabetes mellitus	131 (29%)	80 (23%)	51 (44%)	-20% (-30%; -11%)	<0.001
Current smoker	231 (50%)	184 (54%)	47 (40%)	14% (3%; 24%)	0.01
Hypercholesterolemia	220 (48%)	171 (50%)	49 (42%)	(-2%; 19%)	0.13
Previous myocardial infarction	84 (18%)	60 (18%)	24 (21%)	-3% (-11%; 5%)	0.49
History of malignancy	69 (15%)	57 (17%)	12 (10%)	6% (-1%; 14%)	0.10
Chronic obstructive lung disease	48 (10%)	41 (12%)	7 (6%)	6% (-0%; 12%)	0.08
Clinically relevant valvular disease	22 (5%)	14 (4%)	8 (7%)	-3% (-7%; 2%)	0.22
NYHA class					<0.001
NYHA I	214 (48%)	190 (56%)	24 (23%)	33% (22%; 43%)	<0.001
NYHA II	77 (17%)	61 (18%)	16 (15%)	3% (-6%; 11%)	0.66
NYHA III	70 (16%)	55 (16%)	15 (14%)	2% (-6%; 10%)	0.76
NYHA IV	83 (19%)	34 (10%)	49 (47%)	-37% (-45%; -29%)	<0.001
Cardiogenic shock (Killip 4)	24 (5%)	11 (3%)	13 (11%)	-8% (-13%; -3%)	0.003
Fever	56 (12%)	22 (6%)	34 (30%)	-24% (-30%; -17%)	<0.001
Interventions performed during index hospitalization					
Coronary procedure	247 (54%)	221 (65%)	26 (22%)	42% (33%; 52%)	<0.001
TAVI	0 (0%)	0 (0%)	0 (0%)	0%	

Intervention for major arrhythmia	35 (8%)	22 (6%)	13 (11%)	-5% (-10%; 1%)	0.11
Transfer to Intensive Care Unit during index hospitalization	108 (24%)	48 (14%)	60 (51%)	-37% (-46%; -29%)	<0.001

Depicted are means with standard deviations (±) and counts with percentages of patients with non-missing data (%). P-values from t-tests or Fisher's tests (- or chisquare tests in case more than 2 x 2 comparisons).

NYHA: New York Heart Association; TAVI: transcatheter aortic valve index.

Supplemental Table 4. **Laboratory analyses during index hospitalization of enrolled patients excluding patients with severe aortic stenosis**

	All patients N=459	Controls N=342	Cases N=117	Difference (95% CI)	p Value
**Timepoint 1***					
Leucocytes (G/L)	n = 448, 10.0 ± 4.8	n = 332, 10.1 ± 4.3	n = 116, 9.6 ± 6.2	0.6 (-0.5; 1.6)	0.29
Hemoglobin (g/L)	n = 448, 134.1 ± 23.4	n = 332, 135.9 ± 22.7	n = 116, 128.8 ± 24.8	7.1 (2.1; 12.0)	0.005
Platelets (G/L)	n = 447, 231.2 ± 80.3	n = 332, 236.0 ± 80.4	n = 115, 217.5 ± 78.8	18.5 (1.5; 35.5)	0.03
hs-CRP (mg/L)	n = 306, 52.1 ± 76.0	n = 210, 32.7 ± 60.1	n = 96, 94.4 ± 89.3	-61.7 (-78.8; -44.6)	<0.001
CK (U/L)	n = 421, 419.8 ± 896.4	n = 324, 473.3 ± 990.1	n = 97, 241.1 ± 418.1	232.2 (29.3; 435.1)	0.025
CKMB (µg/L)	n = 199, 67.2 ± 114.1	n = 137, 66.7 ± 105.4	n = 62, 68.4 ± 132.2	-1.7 (-36.1; 32.7)	0.92
hs-TnT (ng/L)	n = 426, 901.8 ± 2750.4	n = 324, 1036.3 ± 2945.2	n = 102, 474.7 ± 1961.8	561.6 (-50.4; 1173.6)	0.07
eGFR (mL/min)	n = 436, 69.7 ± 26.1	n = 331, 71.1 ± 25.0	n = 105, 65.1 ± 28.9	6.1 (0.4; 11.8)	0.04
**Timepoint 2****					
Leucocytes (G/L)	n = 368, 8.7 ± 3.8	n = 262, 8.5 ± 3.0	n = 106, 9.4 ± 5.3	-0.9 (-1.8; -0.1)	0.04
Hemoglobin (g/L)	n = 369, 122.3 ± 23.7	n = 262, 126.2 ± 22.2	n = 107, 12.9 ± 24.9	13.2 (8.0; 18.4)	<0.001
Platelets (G/L)	n = 367, 244.9 ± 100.1	n = 262, 240.2 ± 97.7	n = 105, 256.6 ± 105.4	-16.5 (-39.2; 6.2)	0.15
hs-CRP (mg/L)	n = 227, 39.9 ± 58.0	n = 145, 29.8 ± 38.2	n = 82, 57.7 ± 79.4	-27.9 (-43.3; -12.6)	<0.001
CK (U/L)	n = 362, 790.1 ± 2249.1	n = 290, 761.7 ± 1299.1	n = 72, 904.4 ± 4339.7	-142.6 (-725.2; 439.9)	0.63
CKMB (µg/L)	n = 225, 59.9 ± 110.1	n = 163, 68.7 ± 118.4	n = 62, 36.9 ± 80.7	31.8 (-0.3; 63.9)	0.05
hs-TnT (ng/L)	n = 368, 1631.6 ± 3477.2	n = 291, 1903.1 ± 3723.3	n = 77, 605.4 ± 2034.6	1297.7 (431.0; 2164.4)	0.003
eGFR (mL/min)	n = 396, 68.3 ± 28.3	n = 304, 69.4 ± 27.2	n = 92, 64.7 ± 31.6	4.7 (-1.9; 11.3)	0.16

* Time point closest to admission for index hospitalization

** Time point closest to hospital discharge

CK: creatine kinase; eGFR: estimated glomerular filtration rate; hs-CRP: high-sensitivity C-reactive protein; hs-TnT: high-sensitivity troponin T.

Supplemental Table 5. In-hospital clinical outcomes of enrolled patients excluding patients with severe aortic stenosis

	Controls N=342	Cases N=117	Difference (95% CI)	p-value
Death	4 (1%)	20 (17%)	-16% (-20%; -11%)	<0.001
Cardiovascular death	2 (1%)	4 (3%)	-3% (-5%; -0%)	0.04
Non-cardiovascular death	2 (1%)	15 (13%)	-12% (-16%; -8%)	<0.001
Unclear death	0 (0%)	1 (1%)	-1% (-2%; 0%)	0.25
Myocardial infarction	3 (1%)	3 (3%)	-2% (-4%; 1%)	0.18
Unplanned revascularisation	0 (0%)	0 (0%)	0%	
Cerebrovascular event	0 (0%)	1 (1%)	-1% (-2%; 0%)	0.25
Stroke (ischemic or hemorhagic)	0 (0%)	1 (1%)	-1% (-2%; 0%)	0.25
Acute kidney injury	6 (2%)	10 (9%)	-7% (-11%; -3%)	0.002
Major arrhythmia	15 (4%)	18 (15%)	-11% (-16%; -6%)	<0.001

Depicted are counts (%).

Supplemental Table 6. Clinical outcomes at 30 days and one year* excluding patients with severe aortic stenosis

	Outcomes at 30 days				Outcomes at 1 year			
	Controls N=342	Cases N=117	Subdistribution Hazard ratio (95% CI)	p-value	Controls N=342	Cases N=117	Subdistribution Hazard ratio (95% CI)	p-value
Death	8 (2.4%) [0]	23 (19.8%) [0]	9.13 (4.08-20.44)	<0.001	24 (7.7%) [0]	26 (29.2%) [0]	4.31 (2.53-7.35)	<0.001
Cardiovascular death	3 (0.9%) [5]	5 (4.3%) [18]	4.93 (1.18-20.60)	0.03	9 (2.9%) [15]	5 (5.5%) [21]	1.94 (0.68-5.53)	0.21
Non-cardiovascular death	2 (0.6%) [6]	17 (14.8%) [6]	26.62 (6.16-115.04)	<0.001	9 (2.9%) [15]	19 (20.5%) [7]	7.87 (3.74-16.56)	<0.001
Unclear death	3 (0.9%) [5]	1 (0.9%) [22]	0.97 (0.10-9.28)	0.98	6 (2.0%) [18]	2 (2.3%) [24]	1.17 (0.24-5.73)	0.85
Myocardial infarction	3 (0.9%) [7]	3 (2.6%) [23]	2.95 (0.60-14.53)	0.18	7 (2.2%) [22]	3 (3.3%) [26]	1.47 (0.40-5.43)	0.56
Unplanned revascularisation	0 (0.0%) [8]	0 (0.0%) [23]			0 (0.0%) [24]	1 (1.6%) [26]		
Cerebrovascular event	0 (0.0%) [8]	1 (0.8%) [23]			1 (0.3%) [24]	2 (2.5%) [26]	7.36 (0.70-77.17)	0.10
Stroke (ischemic or hemorrhagic)	0 (0.0%) [8]	1 (0.8%) [23]			0 (0.0%) [24]	2 (2.4%) [26]		
Acute kidney injury	7 (2.1%) [8]	9 (7.8%) [19]	3.88 (1.45-10.37)	0.007	8 (2.5%) [23]	12 (11.4%)[22]	4.86 (1.97-11.99)	<0.001
Major arrhythmia	16 (4.7%) [7]	18 (15.4%)[18]	3.47 (1.78-6.76)	<0.001	22 (6.8%) [22]	22 (21.3%)[21]	3.42 (1.90-6.17)	<0.001
Hospitalization for heart failure	3 (0.9%) [8]	4 (3.5%) [23]	3.94 (0.88-17.60)	0.07	17 (5.8%) [22]	5 (6.4%) [26]	1.12 (0.42-2.97)	0.82

* First events of each event type under competing risk with death.

Depicted are counts with the Aalen-Johanssen estimator of the cumulative incidence (%) [in square brackets the number of patients with the competing event occurring before the event of interest].

Subdistribution ratio of the hazards using the Fine & Grey methodology with 95 % confidence interval (CI) and p-value comparing controls vs. cases.

Supplemental Table 7. **Baseline characteristics of enrolled SARS-COV-2-positive patients stratified by diabetic status at baseline**

	All cases N = 122	Diabetic N = 54	Non-diabetic N = 68	Difference (95% CI)	p Value
Age (years)	67.6 ± 14.8	71.9 ± 8.4	64.2 ± 17.7	7.7 (2.5; 12.9)	0.004
Male sex	92 (75%)	41 (76%)	51 (75%)	1% (-15%; 17%)	1.000
Diagnosis at index hospitalization					0.28
Acute coronary syndrome	32 (26%)	14 (26%)	18 (26%)	-1% (-17%; 15%)	1.00
New-onset major arrhythmia	38 (31%)	12 (22%)	26 (38%)	-16% (-33%; 1%)	0.08
Acute heart failure	43 (35%)	24 (44%)	19 (28%)	17% (-1%; 34%)	0.08
Symptomatic severe aortic stenosis	5 (4%)	3 (6%)	2 (3%)	3% (-5%; 10%)	0.65
Hospitalization related to adult congenital heart disease	1 (1%)	0 (0%)	1 (1%)	-1% (-5%; 2%)	1.00
Clinically suspected myocarditis	3 (2%)	1 (2%)	2 (3%)	-1% (-7%; 5%)	1.00
Diabetes mellitus					
Current smoker	50 (41%)	26 (48%)	24 (35%)	13% (-5%; 31%)	0.19
Hypercholesterolemia	54 (44%)	31 (57%)	23 (34%)	24% (6%; 41%)	0.01
Previous myocardial infarction	26 (21%)	16 (30%)	10 (15%)	15% (0%; 30%)	0.08
History of malignancy	13 (11%)	9 (17%)	4 (6%)	11% (-0%; 22%)	0.08
Chronic obstructive lung disease	8 (7%)	3 (6%)	5 (7%)	-2% (-11%; 7%)	1.00
Clinically relevant valvular disease	11 (9%)	7 (13%)	4 (6%)	7% (-3%; 17%)	0.21
NYHA class					0.24
NYHA I	24 (22%)	7 (14%)	17 (29%)	-15% (-30%; 1%)	0.07
NYHA II	18 (17%)	8 (16%)	10 (17%)	-1% (-15%; 13%)	1.00
NYHA III	17 (16%)	10 (20%)	7 (12%)	8% (-6%; 22%)	0.29
NYHA IV	50 (46%)	25 (50%)	25 (42%)	8% (-11%; 27%)	0.45
Cardiogenic shock (Killip 4)	13 (11%)	n = 54, 6 (11%)	n = 68, 7 (10%)	1% (-10%; 12%)	1.00
Fever	34 (29%)	18 (35%)	16 (24%)	10% (-6%; 27%)	0.23
Interventions performed during index hospitalization					
Coronary procedure	26 (21%)	10 (19%)	16 (24%)	-5% (-20%; 10%)	0.66
TAVI	2 (2%)	2 (4%)	0 (0%)	4% (-1%; 8%)	0.19

Intervention for major arrhythmia	13 (11%)	5 (9%)	8 (12%)	-3% (-14%; 9%)	0.77
Transfer to Intensive Care Unit during index hospitalization	60 (49%)	27 (50%)	33 (49%)	1% (-17%; 20%)	1.00

Depicted are means with standard deviations (±) and counts with percentages of patients with non-missing data (%). P-values from t-tests or Fisher's tests (- or chisquare tests in case more than 2 x 2 comparisons).

NYHA: New York Heart Association; TAVI: transcatheter aortic valve index.

Supplemental Table 8. Laboratory analyses during index hospitalization of enrolled SARS-COV-2-positive patients stratified by diabetic status at baseline

	All cases<br>N = 122	Diabetic<br>N = 54	Non-diabetic<br>N = 68	Difference (95% CI)	p Value
**Timepoint 1***					
Leucocytes (G/L)	n = 120, 9.5 ± 6.1	n = 53, 9.1 ± 5.0	n = 67, 9.7 ± 6.9	-0.7 (-2.9; 1.6)	0.55
Hemoglobin (g/L)	n = 120, 128.7 ± 24.7	n = 53, 122.9 ± 26.1	n = 67, 133.3 ± 22.7	-10.4 (-19.2; -1.6)	0.02
Platelets (G/L)	n = 119, 216.4 ± 78.1	n = 53, 220.6 ± 75.4	n = 66, 213.1 ± 80.7	7.5 (-21.1; 36.1)	0.60
hs-CRP (mg/L)	n = 97, 93.5 ± 89.3	n = 41, 107.9 ± 88.8	n = 56, 83.0 ± 88.9	24.8 (-11.3; 61.0)	0.18
CK (U/L)	n = 102, 251.3 ± 443.5	n = 44, 220.0 ± 327.6	n = 58, 275.1 ± 516.0	-55.1 (-231.1; 121.0)	0.54
CKMB (µg/L)	n = 63, 67.6 ± 131.3	n = 27, 58.5 ± 116.8	n = 36, 74.4 ± 142.4	-15.9 (-82.4; 50.7)	0.64
hs-TnT (ng/L)	n = 106, 458.7 ± 1925.8	n = 46, 146.5 ± 226.1	n = 60, 698.1 ± 2535.1	-551.6 (-1294.6; 191.4)	0.14
eGFR (mL/min)	n = 109, 64.2 ± 29.1	n = 45, 51.5 ± 29.2	n = 64, 73.2 ± 25.6	-21.7 (-32.1; -11.2)	<0.001
**Timepoint 2****					
Leucocytes (G/L)	n = 111, 9.2 ± 5.3	n = 48, 8.5 ± 4.8	n = 63, 9.8 ± 5.6	-1.3 (-3.3; 0.7)	0.20
Hemoglobin (g/L)	n = 112, 113.3 ± 25.0	n = 49, 109.7 ± 21.2	n = 63, 116.2 ± 27.4	-6.5 (-15.9; 2.9)	0.17
Platelets (G/L)	n = 110, 253.2 ± 104.9	n = 48, 243.5 ± 107.8	n = 62, 260.7 ± 102.8	-17.2 (-57.1; 22.8)	0.40
hs-CRP (mg/L)	n = 85, 56.3 ± 78.4	n = 32, 53.1 ± 76.2	n = 53, 58.2 ± 80.3	-5.1 (-40.0; 29.8)	0.77
CK (U/L)	n = 76, 884.5 ± 4227.6	n = 35, 1326.5 ± 6172.0	n = 41, 507.2 ± 902.5	819.2 (-1110.6; 2749.0)	0.40
CKMB (µg/L)	n = 63, 36.6 ± 80.0	n = 28, 27.9 ± 63.5	n = 35, 43.6 ± 91.4	-15.8 (-56.1; 24.5)	0.44
hs-TnT (ng/L)	n = 80, 585.1 ± 1998.3	n = 33, 229.1 ± 470.7	n = 47, 835.1 ± 2559.1	-606.0 (-1499.9; 287.9)	0.18
eGFR (mL/min)	n = 96, 63.7 ± 31.7	n = 40, 48.6 ± 28.9	n = 56, 74.5 ± 29.4	-25.9 (-37.8; -13.9)	<0.001

* Time point closest to admission for index hospitalization

** Time point closest to hospital discharge

CK: creatine kinase; eGFR: estimated glomerular filtration rate; hs-CRP: high-sensitivity C-reactive protein; hs-TnT: high-sensitivity troponin T.

Supplemental Table 9. In-hospital clinical outcomes of enrolled SARS-COV-2-positive patients stratified by diabetic status at baseline

	Diabetic N = 54	Non-diabetic N = 68	Difference (95% CI)	p-value
Death	9 (17%)	11 (16%)	0%	1.00
Cardiovascular death	1 (2%)	3(4%)	-3% (-9%; 4%)	0.63
Non-cardiovascular death	7 (13%)	8 (12%)	1% (-11%; 13%)	1.00
Unclear death	1 (2%)	0 (0%)	2% (-1%; 5%)	0.44
Myocardial infarction	1(2%)	2 (3%)	-1% (-7%; 5%)	1.00
Unplanned revascularisation	0 (0%)	0 (0%)	0%	
Cerebrovascular event	0 (0%)	1 (1%)	-1% (-5%; 2%)	1.00
Stroke (ischemic or hemorhagic)	0 (0%)	1 (1%)	-1% (-5%; 2%)	1.00
Acute kidney injury	7 (13%)	3 (4%)	9% (-1%; 18%)	0.11
Major arrhythmia	7 (13%)	11 (16%)	-3% (-16%; 10%)	0.80

Depicted are counts (%).

Supplemental Table 10. **Clinical outcomes at 30 days and one year* of enrolled SARS-COV-2-positive patients stratified by diabetic status at baseline**

	**Outcomes at 30 days**				**Outcomes at 1 year**			
	Diabetic N = 54	Non-diabetic N = 68	Subdistribution Hazard ratio (95% CI)	p-value	Diabetic N = 54	Non-diabetic N = 68	Subdistribution Hazard ratio (95% CI)	p-value
Death	12 (22.1%) [0]	11 (16.6%) [0]	1.37 (0.61-3.09)	0.45	14 (27.3%) [0]	12 (19.4%) [0]	1.48 (0.69-3.17)	0.32
Cardiovascular death	2 (3.7%) [10]	3 (4.5%) [8]	0.84 (0.14-4.99)	0.84	2 (3.7%) [12]	3 (4.5%) [9]	0.84 (0.14-4.99)	0.84
Non-cardiovascular death	9 (16.6%) [3]	8 (12.1%) [3]	1.41 (0.55-3.63)	0.48	10 (19.6%) [4]	9 (14.5%) [3]	1.40 (0.57-3.41)	0.46
Unclear death	1 (1.9%) [11]	0 (0.0%) [11]			2 (4.6%) [12]	0 (0.0%) [12]		
Myocardial infarction	1 (1.9%) [12]	2 (2.9%) [11]	0.63 (0.06-6.92)	0.70	1 (1.9%) [14]	2 (2.9%) [12]	0.63 (0.06-6.92)	0.70
Unplanned revascularisation	0 (0.0%) [12]	0 (0.0%) [11]			0 (0.0%) [14]	1 (2.9%) [12]		
Cerebrovascular event	0 (0.0%) [12]	1 (1.5%) [11]			1 (2.7%) [14]	1 (2.1%) [12]	1.25 (0.08-19.52)	0.87
Stroke (ischemic or hemorrhagic)	0 (0.0%) [12]	1 (1.5%) [11]			1 (2.7%) [14]	1 (2.1%) [12]	1.25 (0.08-19.52)	0.87
Acute kidney injury	6 (11.0%) [10]	3 (4.4%) [9]	2.57 (0.65-10.16)	0.18	9 (19.0%) [12]	3 (5.2%) [10]	3.93 (1.06-14.53)	0.04
Major arrhythmia	7 (12.9%) [11]	11 (16.1%) [7]	0.79 (0.31-2.02)	0.62	8 (17.6%) [13]	14 (23.9%) [8]	0.71 (0.30-1.67)	0.43
Hospitalization for heart failure	2 (3.8%) [12]	2 (3.0%) [11]	1.24 (0.18-8.79)	0.83	2 (4.9%) [14]	3 (5.5%) [12]	0.88 (0.15-5.09)	0.89

* First events of each event type under competing risk with death.

Depicted are counts with the Aalen-Johanssen estimator of the cumulative incidence (%) [in square brackets the number of patients with the competing event occurring before the event of interest].

Supplemental Table 11. **Baseline characteristics of enrolled SARS-COV-2-positive patients in relation to Intensive Care Unit transfer or not during index hospitalization**

	All cases<br>N = 122	No ICU transfer<br>N = 62	ICU transfer<br>N = 60	Difference (95% CI)	p Value
Age (years)	67.6 ± 14.8	65.9 ± 15.7	69.4 ± 13.7	-3.6 (-8.9; 1.7)	0.12
Male sex	92 (75%)	50 (81%)	42 (70%)	11% (-5%; 26%)	0.21
Diagnosis at index hospitalization					0.14
Acute coronary syndrome	32 (26%)	14 (23%)	18 (30%)	-7% (-23%; 8%)	0.41
New-onset major arrhythmia	38 (31%)	22 (35%)	16 (27%)	9% (-8%; 25%)	0.33
Acute heart failure	43 (35%)	19 (31%)	24 (40%)	-9% (-27%; 8%)	0.34
Symptomatic severe aortic stenosis	5 (4%)	5 (8%)	0 (0%)	8% (1%; 15%)	0.06
Hospitalization related to adult congenital heart disease	1 (1%)	1 (2%)	0 (0%)	2% (-2%; 5%)	1.00
Clinically suspected myocarditis	3 (2%)	1 (2%)	2 (3%)	-2% (-7%; 4%)	0.62
Diabetes mellitus	54 (44%)	27 (44%)	27 (45%)	-1% (-19%; 17%)	1.00
Current smoker	50 (41%)	22 (35%)	28 (47%)	-11% (-29%; 6%)	0.27
Hypercholesterolemia	54 (44%)	32 (52%)	22 (37%)	15% (-3%; 33%)	0.10
Previous myocardial infarction	26 (21%)	15 (24%)	11 (18%)	6% (-9%; 21%)	0.51
History of malignancy	13 (11%)	6 (10%)	7 (12%)	-2% (-13%; 9%)	0.78
Chronic obstructive lung disease	8 (7%)	5 (8%)	3 (5%)	3% (-6%; 12%)	0.72
Clinically relevant valvular disease	11 (9%)	8 (13%)	3 (5%)	8% (-2%; 18%)	0.20
NYHA class					0.24
NYHA I	24 (22%)	15 (27%)	9 (17%)	10% (-6%; 26%)	0.25
NYHA II	18 (17%)	15 (27%)	3 (6%)	21% (7%; 35%)	0.004
NYHA III	17 (16%)	10 (18%)	7 (13%)	5% (-9%; 19%)	0.60
NYHA IV	50 (46%)	16 (29%)	34 (64%)	-36% (-53%; -18%)	<0.001
Fever	34 (29%)	15 (24%)	19 (34%)	-10% (-26%; 7%)	0.31
Interventions performed during index hospitalization					
Coronary procedure	26 (21%)	0 (16%)	16 (27%)	-11% (-25%; 4%)	0.19

TAVI	2 (2%)	2 (3%)	0 (0%)	3% (-1%; 8%)	0.49

Depicted are means with standard deviations (±) and counts with percentages of patients with non-missing data (%). P-values from t-tests or Fisher's tests (- or chisquare tests in case more than 2 x 2 comparisons).

NYHA: New York Heart Association; TAVI: transcatheter aortic valve index.

Supplemental Table 12. **Laboratory analyses during index hospitalization of enrolled SARS-COV-2-positive patients in relation to Intensive Care Unit transfer or not during index hospitalization**

	All cases N = 122	No ICU transfer N = 62	ICU transfer N = 60	Difference (95% CI)	p Value
**Timepoint 1***					
Leucocytes (G/L)	9.5 ± 6.1	8.5 ± 4.9	10.4 ± 7.0	-2.0 (-4.2; 0.2)	0.07
Hemoglobin (g/L)	128.7 ± 24.7	128.1 ± 28.7	129.3 ± 20.1	-1.2 (-10.2; 7.7)	0.79
Platelets (G/L)	216.4 ± 78.1	204.2 ± 72.7	228.5 ± 82.0	-24.3 (-52.5; 3.8)	0.09
hs-CRP (mg/L)	93.5 89.3	64.1 ± 72.5	121.2 ± 95.2	-57.0 (-91.2; -22.8)	0.001
CK (U/L)	251.3 ± 443.5	287.6 ± 528.1	222.7 ± 365.7	64.9 (-110.6; 240.3)	0.47
CKMB (µg/L)	67.6 ± 131.3	85.2 ± 132.2	60.0 ± 131.7	25.2 (-46.4; 96.8)	0.49
hs-TnT (ng/L)	458.7 ± 1925.8	337.3 ± 1036.1	555.4 ± 2416.8	-218.0 (-965.8; 529.7)	0.56
eGFR (mL/min)	64.2 ± 29.1	67.3 ± 30.2	61.1 ± 27.8	6.3 (-4.8; 17.3)	0.26
**Timepoint 2****					
Leucocytes (G/L)	9.2 ± 5.3	7.7 ± 3.9	10.6 ± 6.0	-2.9 (-4.8; -1.0)	0.003
Hemoglobin (g/L)	113.3 ± 25.0	122.6 ± 25.9	104.7 ± 20.8	17.9 (9.1; 26.6)	<0.001
Platelets (G/L)	253.2 ± 104.9	231.6 ± 95.5	272.5 ± 109.9	-40.9 (-80.0; -1.8)	0.04
hs-CRP (mg/L)	56.3 ± 78.4	38.4 ± 57.5	70.0 ± 89.4	-31.5 (-65.0; 1.9)	0.06
CK (U/L)	884.5 ± 4227.6	356.4 ± 565.7	1143.4 ± 5142.7	-786.9 (-2836.0; 1262.1)	0.45
CKMB (µg/L)	36.6 ± 80.0	35.8 ± 73.3	36.9 ± 82.9	-1.1 (-47.3; 45.1)	0.96
hs-TnT (ng/L)	585.1 ± 1998.3	647.0 ± 1806.3	551.8 ± 2110.7	95.2 (-837.8; 1028.1)	0.84
eGFR (mL/min)	63.7 ± 31.7	70.2 ± 30.2	58.1 ± 32.2	12.1 (-0.6; 24.8)	0.06

* Time point closest to admission for index hospitalization

** Time point closest to hospital discharge

CK: creatine kinase; eGFR: estimated glomerular filtration rate; hs-CRP: high-sensitivity C-reactive protein; hs-TnT: high-sensitivity troponin T.
